# Correction: Inferring school district learning modalities during the COVID-19 pandemic with a hidden Markov model

**DOI:** 10.1371/journal.pone.0300861

**Published:** 2024-03-14

**Authors:** Mark J. Panaggio, Mike Fang, Hyunseung Bang, Paige A. Armstrong, Alison M. Binder, Julian E. Grass, Jake Magid, Marc Papazian, Carrie K. Shapiro-Mendoza, Sharyn E. Parks

There are errors in the author affiliations. The correct affiliations are as follows:

Mark J. Panaggio^1^, Mike Fang^1^, Hyunseung Bang^1^, Paige A. Armstrong^2^, Alison M. Binder^2^, Julian E. Grass^2^, Jake Magid^3^, Marc Papazian^3^, Carrie K. Shapiro-Mendoza^2^, Sharyn E. Parks^2^

**1** Johns Hopkins University Applied Physics Laboratory, Laurel, Maryland, USA, **2** COVID-19 Response Team, Centers for Disease Control and Prevention, Atlanta, Georgia, USA, **3** Palantir Technologies, Denver, Colorado, USA.
